# A Siamese Network-Based Non-Contact Measurement Method for Railway Catenary Uplift Trained in a Free Vibration Test

**DOI:** 10.3390/s20143984

**Published:** 2020-07-17

**Authors:** Fuchuan Duan, Zhigang Liu, Donghai Zhai, Anders Rønnquist

**Affiliations:** 1 State Key Laboratory of Traction Power and School of Electrical Engineering, Southwest Jiaotong University, Chengdu 610031, China; bk20093978@my.swjtu.edu.cn (F.D.); liuzg@swjtu.cn (Z.L.); 2 School of Information Science and Technology, Southwest Jiaotong University, Chengdu 610031, China; 3 Department of Structural Engineering, Norwegian University of Science and Technology, 7491 Trondheim, Norway; anders.ronnquist@ntnu.no

**Keywords:** electrified railway, catenary free vibration, non-contact measurement, siamese neural network, object tracking

## Abstract

The vibration of the catenary that is initiated by the passing pantograph has a direct influence on the pantograph–catenary contact performance. Monitoring the dynamic uplift of the catenary can help inspectors to evaluate the railway operation conditions and investigate the mechanism of pantograph–catenary interaction further. In this paper, a non-contact measurement method based on the deep leaning method is proposed to monitor the real-time vibration of the catenary. The field test for the catenary free vibration is designed to validate the method’s performance. The measurement method is developed based on the fully convolutional Siamese neural network, and the contact wire is taken as the tracking target. To reduce the recognition errors caused by the changes in the shape and grayscale of the moving contact wire in images, the class-agnostic binary segmentation mask is adopted. A developed down-sampling block is used in the neural network to reduce the image feature loss, which effectively enhances the recognition effect for the catenary vibration under variable lighting conditions. To validate the performance of the proposed measurement method, a series of field tests of catenary free vibration were conducted under various lighting conditions and different excitations, and the recognition results were compared with traditional target tracking methods. The results show that the proposed method performs well for catenary vibration identification in the field test. Additionally, the uplift data extracted from the identified images agree with the numerical results, and also help to further investigate the wave propagation and damping characteristics in the catenary structure.

## 1. Introduction

**Figure 1:**
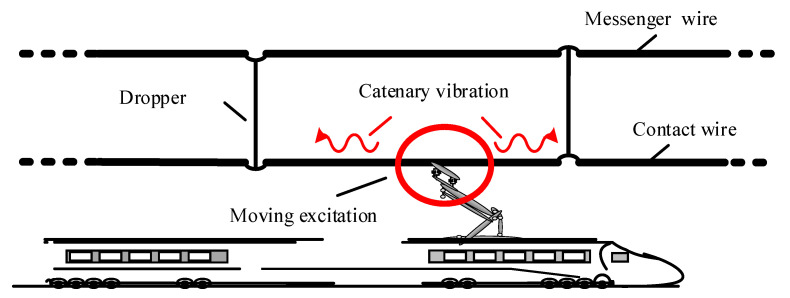
Schematic of the pantograph-catenary system.

The sliding contact between the pantograph and the catenary is the only source of power for electric trains, and directly determines the normal operation of electrified railways without traffic interruptions. The catenary system, as a structure comprised of tension cables and hangers, has a high flexibility, and exhibits complex vibrations under the excitation of moving pantographs, especially when it is subjected to complex environmental disturbances [1]. As shown in Figure 1, the travelling wave propagating in the catenary causes complex vibration behaviors of the catenary. It is stated in the European railway standard EN 50367 [2] that the direct reflection of the current collection quality is the contact force between the registration strip of the pantograph and the contact wire. Normally, several contact force statistics, such as the mean value, standard deviation, and maximum value, are used to check the acceptance of the interaction performance of the pantograph-catenary. However, due to the limitation of the measurement device, the frequency of interest of the contact force is restricted to 0–20 Hz [3], which results in the loss of important high-frequency information. In addition to the contact force, the maximum uplift of the contact wire is also included to evaluate the interaction performance, and is strictly limited to ensure that the pantograph cannot damage the catenary. This requires an in-time and precise measurement of the contact wire vibration.

However, previous studies on the pantograph-catenary interaction have mainly relied on numerical modeling, such as the computation of the initial configuration [4,5,6], the optimization of structural parameters [7], the inclusion of environmental perturbations [1,8,9,10,11,12,13,14], and the development of efficient numerical algorithms [15,16,17,18,19]. Although numerical models are beneficial for improving the understanding of pantograph–catenary dynamics, calculation errors caused by the simplification to complex operation conditions are common challenges to the numerical accuracy. Therefore, the implementation of field measurement is necessary, as it provides real-life data for the further modification and improvement of numerical techniques.

With the development of detection technology, many methods have been applied in condition monitoring of the catenary system. Currently, there are two main methods for pantograph–catenary condition measurement: Non-contact methods based on image processing and contact-type schemes based on sensors installed in the system. In previous years, contact-type schemes have been widely used in railway condition detection, such as contact force collection [20,21] and acceleration collection [22,23]. However, these approaches require parts of the measurement system to be installed on the catenary structure, which means that the railway traffic will be interrupted and measurement will take a more substantial amount of time. Compared with contact-type methods, non-contact approaches do not introduce additional masses or foreign matters which may disturb the dynamic or aerodynamic behaviors. Besides that, the non-contact methods are also always much more convenient to install and barely have an influence on the railway traffic. Recently, with the great progress of computer vision techniques and relevant inspection equipment, image-based techniques have become the most promising approaches in state inspections of the railway system. For instance, vision-based systems have been widely used in catenary geometry parameter detection [24,25,26], the abnormal behavior localization of pantograph–catenary contact [27], and catenary support fault localization [28].

The image-based technique has also been adopted in catenary vibration measurement in the literature. Zou et al. [29] proposed a set of photogrammetric devices in their work to obtain the displacement histories of a catenary wire in the laboratory. The non-contact inspection approach has been proven to be effective in catenary vibration identification. However, the general problem in catenary uplift measurement has still not been well-addressed, which is that tracking the contact wire is challenging when images are taken with busy backgrounds. The reasons for this are as follows: (1) Most of the existing schemes use artificial methods to weaken the interference of backgrounds, such as taking pure sky as the background and hanging a white board behind cables [30]. The measurement challenge caused by the operation conditions of railways has still not been addressed. Additionally, (2) most current works focus on classical image processing techniques, such as edge detection methods, which lack robustness in contact wire tracking under non-ideal conditions, and may even fail to accurately detect catenary vibration.

According to the discussion above, this paper intends to propose an improved measurement method for catenary vibration. The proposed detection approach is developed based on a high-speed CCD camera and relevant devices. Compared with the presented works on catenary vibration detection, the measurement method proposed in this paper takes advantage of the promising deep learning techniques in vibrating contact wire localization. Moreover, the effectiveness of the method is tested on a real catenary in a field environment. The main contributions are as follows: (1) A measurement method based on the deep learning algorithm is proposed for catenary vibration detection, and the identification performance under different vibration conditions in a field environment is analyzed to show the effectiveness; (2) given that the image features change with catenary vibration and lighting conditions, an improved down-sampling block is applied to reduce the image feature loss, with the mask branch being used to improve the segmentation results; (3) the minimum enclosing rectangle method is adopted to eliminate the error caused by the changing cable shape; and (4) the consistency of the measurement results and the simulation results is verified, through which the value of the proposed method in catenary dynamics research is demonstrated.

**Figure 2:**
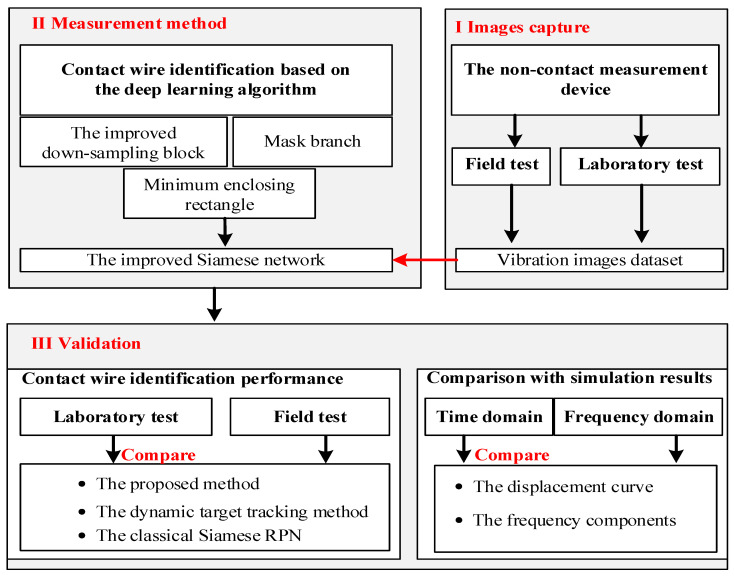
Main ideas and their relations in this paper.

The main ideas and the organization of this paper are summarized in Figure 2. Main ideas and their relations in this paper are divided into three parts, including the image capture part, the measurement method part, and the validation part. In Section 2, a set of photogrammetric devices are designed to obtain the catenary vibration images. Then, an object tracking method based on the neural network is presented in Section 3, considering the detection difficulty under non-ideal conditions when carried out in the field. In Section 4, a series of analyses are conducted to verify the measurement results, including the image recognition performance and data features in the frequency domain.

## 2. A Photogrammetric Device for Catenary Uplift Detection

**Figure 3:**
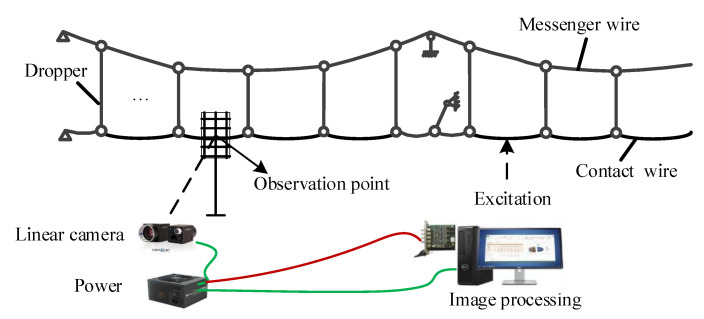
Structural schematic of the non-contact measurement devices employed for catenary vibration.

Vision technology has been widely used in the research field of railway engineering. In this paper, based on vision technology, a set of photogrammetric devices are developed for contact wire vibration measurement, with the aim of providing an approach for the further study of catenary fluctuation. As shown in Figure 3, the measurement system consists of three parts: a high-speed linear camera (i.e., the photogrammetric device) for acquiring images of the moving contact wire, the image processing system, and the actual catenary system (i.e., the measurement object). The camera is positioned on the tripod, a computer is placed on the nearby platform, and an Ethernet cable is used to connect the camera and the image processing system to the computer.

**Figure 4:**
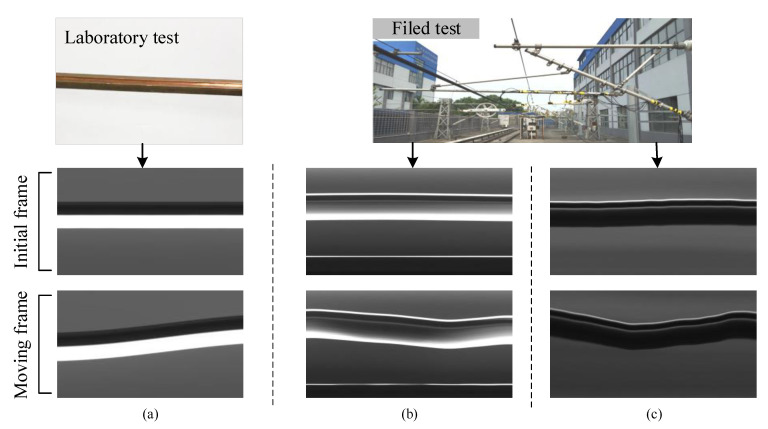
Contact wire vibration images obtained from the non-contact measurement system. (**a**) Contact wire images obtained from the laboratory test with an illumination supplement. (**b**) Contact wire images obtained under strong sunlight. (**c**) Contact wire images obtained under weak sunlight.

The workflow of the camera is given as follows. First, a static image of the contact wire is captured. Second, a transient impact simulating the pantograph contact is applied at the excitation point. Finally, the image of the moving contact wire is captured and transmitted for the next data processing step. Figure 4 presents the cable vibration images obtained from the same set of devices when applied in a laboratory experiment and a field experiment, respectively. As shown in Figure 4, in contrast to the laboratory experiment, the grayscale value of the obtained images changes dramatically without the illumination supplement from the LED lighting source. Additionally, the shape change caused by the cable motion makes it harder to measure the cable vibration accurately.

## 3. Target Tracking Method

The field measurement for catenary vibration in this paper is expected to obtain the catenary vertical displacement under different impacts, which can be calculated by the continuous coordinates of the cable vibration images. However, the change of the grayscale value and the cable shape shown in Figure 4 may lead to data loss. The traditional nonlinear dynamic tracking method based on a combination tracker was designed to improve the measurement accuracy at the very beginning, but it was proved to lack robustness and accuracy.

In this paper, a non-contact measurement method based on deep learning is developed to acquire the catenary vertical vibration in a field environment. The contact wire is taken as the tracking object in the measurement scheme, and an additional class-agnostic binary segmentation mask branch and improved down-sampling block are then adopted into the network to reduce the recognition errors caused by the cable movement and illumination variation. The details of the measurement method are described in the following section.

### 3.1. Architecture of the Network

**Figure 5:**
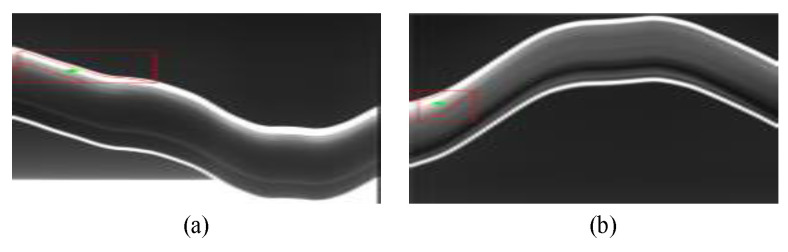
Position recognition of the contact wire based on the nonlinear tracking method. (**a**) The 18th frame. (**b**) The 28th frame.

**Figure 6:**
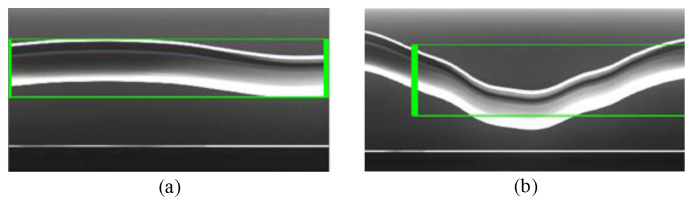
Position recognition of the contact wire based on the classical Siamese region proposal network (RPN). (**a**) The 8th frame. (**b**) The 91st frame.

Although the traditional nonlinear tracking method is easy realize, the robustness of it is still a problem that restricts its application in field tests, as shown in Figure 5. In past decades, the convolution neural network has achieved impressive improvements to become the most widely used approach in many respects. In this paper, inspired by the previous nonlinear tracking method, the fully convolutional Siamese neural network is used to track the moving contact wire instead. The Siamese neural network consists of twin networks, which accept distinct inputs, but are joined by an energy function at the top. Bertinetto et al. [31] proposed an offline trained fully convolutional Siamese network for object tracking, and Li et al. [32] improved it by adding a region proposal network, which is also called RPN. The improved Siamese network is able to predict the position of the target with a bounding box, and outputs the box predictions in parallel with the coordinate axis. However, the classical Siamese RPN network still cannot handle the catenary vibration measurement satisfactorily. The bounding box fails to cover the contact wire in the target when the cable does not have a regular shape, as shown in Figure 6.

**Figure 7:**
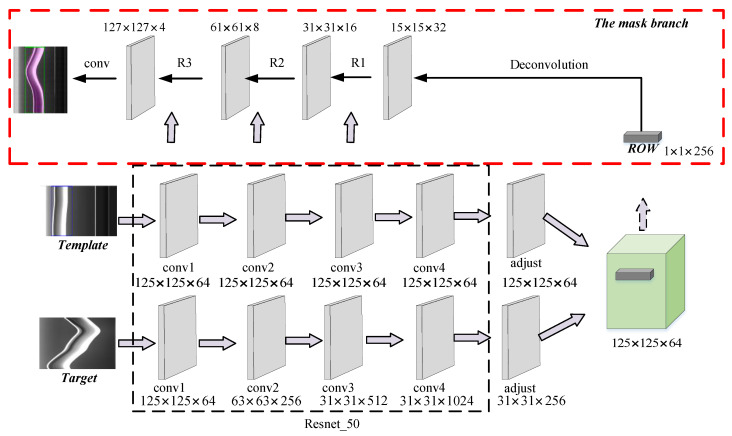
The architecture of the Siamese neural network.

He et al. [33] proposed a useful framework for object instance segmentation that detects objects while simultaneously generating a segmentation mask of each instance. The additional mask branch is used to predict the object mask in parallel with the existing branch for bounding box recognition, which presents the possibility to solve the position problems in contact wire vibration measurement. In this paper, the Siamese network is utilized for catenary vibration measurement. To make the algorithm more robust and improve the measurement accuracy, the mask branch is adopted and the improved down-sampling block is applied in the network architecture to reduce the information loss during image processing. Additionally, the box generation method named the minimum bounding rectangle is also utilized to address the challenge caused by the shape change in the taken images. The architecture of the neural network is shown in Figure 7. The box branch and score branch are set as in the classical RPN network.

### 3.2. Details of the Contact Wire Position Tracking

#### 3.2.1. Backbone

**Figure 8:**
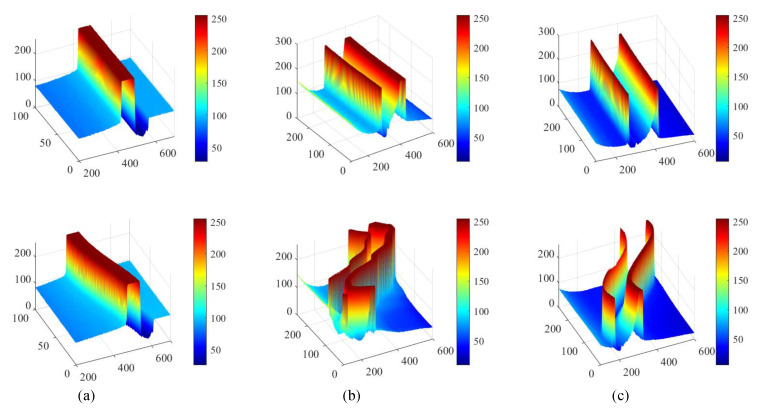
Gray distribution of the contact wires during different motions presented in Figure 4. (**a**) Contact wire images obtained from the laboratory test. (**b**) Contact wire images obtained under strong sunlight. (**c**) Contact wire images obtained under poor sunlight.

Various architectures have been proposed in image processing works, including the Resnet [34], the beamforming prediction network (BPnet) [35], the deep convolutional neural networks (DCNNs) [36], the Alexnet [37]. In these papers, a widely used implementation of four-layer Resnet-50 is adopted as the backbone of the network. The output stride is reduced to 8 to obtain a higher spatial resolution in deep layers. As shown in Figure 4, the gray-level distribution of the taken images always changes with the cable motion, which is related to the variable conditions of the light in the field. Figure 8 depicts the corresponding gray distribution of images shown in Figure 4. It can be seen that the grayscale value changes obviously with the cable vibration, especially at the cable edges. Therefore, it is necessary to maintain the information of the original images as much as possible to make cable identification easier.

**Figure 9:**
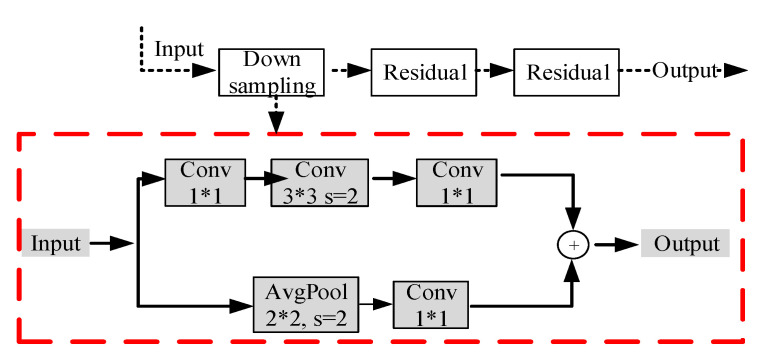
The improved down-sampling block employed in the Resnet network.

The Resnet network consists of an input stem, four subsequent stages, and a final output layer, and detailed information on this network can be found in Table 1 [38]. Different from the classical architecture, after adjusting the channels with the 1×1 kernel, the depth-wise cross-correlation is utilized to produce the multi-channel response map, which can help encode much richer information. For the Resnet architecture, the down-sampling block composed of path A and path B is a vital part, with which every convolutional stage begins. There are three convolutions in path A, with the kernel sizes of 1×1, 3×3, and 1×1, respectively. The first convolution is used to halve the input height and width with a stride of 2. The 1×1 convolution in path B can transform the input shape to the output shape of path A with a stride of 2. Then, the output of the down-sampling block can be obtained by summing the outputs of both paths. Three-quarters of the input feature map are ignored in the original Resnet architecture because of the stride size used in the down-sampling block [34]. In this work, a Resnet tweak is adopted to reduce the information loss, in which the size of the stride of the first two convolutions in path A is switched. Then, the output shape of path A remains unchanged with the kernel size 3×3 in the second convolution. The 1×1 convolution in path B also leads to a 3/4 loss of the input feature maps. To solve that, an 2×2 average pooling layer with the stride of 2 is added to path B of the down-sampling block, and the stride in the following convolution is changed to 1, as shown in Figure 9.

#### 3.2.2. Mask Branch

In contrast to most of the existing tracking methods that rely on low-fidelity object representations, in this paper, the importance of the per-frame binary segmentation masks is discussed. The RoW in the Siamese network can be used not only to output the similarity scores and the bounding box coordinates, but also to encode the image information to produce the pixel-wise binary mask. Then, the Siamese network can be extended with an extra mask branch and the corresponding loss.

The binary mask can be obtained with a two-layer neural network $m_{\kappa }$. Denoting $M_{n}$ as the mask corresponding to the n-th RoW, it can be expressed as follows:

(1) $$M_{n}=m_{\kappa }\left[f_{\theta }\left(x\right)*f_{\theta }\left(z\right)\right],$$

in which $f_{\theta }\left(\cdot \right)$ is the processing procedure conducted by the Resnet network and κ is the learning parameter in the two-layer network. It can be seen that the predicted mask is a function of both the target object in z and the image to segment x. Therefore, for any different reference image, the network can produce a different corresponding segmentation mask.

#### 3.2.3. Loss Function

In the training process, a ground-truth mask $c_{n}$ of size w×h is associated with each RoW, i.e., the region of the candidate window, which is labeled with $y_{n}$ ($y_{n}\in \left\{\pm 1\right\}$). Assuming that the pixel of the object mast in the n-th candidate ROW is pixel (i,j), the corresponding label can be denoted as $c_{n}^{ij}$ ($c_{n}^{ij}\in \left\{\pm 1\right\}$). Then, the loss function of the mask branch can be expressed as follows:

(2) $$L_{mask}=\sum \left(\frac{1+y_{n}}{2wh}\sum \log\left(1+e^{-c_{n}^{ij}m_{n}^{ij}}\right)\right),$$

where i and j are the pixel coordinates, respectively, n is the candidate *RoW* number; and $m_{n}$ is the predicted mask, and $L_{mask}$ is only associated with the positive *RoW*, i.e., $y_{n}=1$.

In this paper, a three-branch Siamese network is used, i.e., the mask branch, the score branch, and the box branch. The box branch and score branch in the Siamese network are trained using the smooth $L_{1}$ and the cross-entropy losses, which are the same as in Li’s work [32]. The loss function of the box branch $L_{box}$ can be expressed as follows:

(3) $$L_{box}=\sum_{i}smooth_{L1}\left(\delta \left(i\right),\sigma \right)\;smooth_{L1}\left(\delta \left(i\right),\sigma \right)=\left\{ \begin{array}{l} 0.5\sigma^{2}\delta^{2}\left(i\right),\left|\delta \left(i\right)\right|<\frac{1}{\sigma^{2}}\\ \left|\delta \left(i\right)\right|-\frac{1}{2\sigma^{2}},\left|\delta \left(i\right)\right|\geq \frac{1}{\sigma^{2}} \end{array}\right.,$$

where σ is the smooth parameter with the value of 3 and δ is the normalized difference between the predicted boxes and the ground-truth boxes.

Then, the loss function of the three-branch network is defined as

(4) $$L=a\cdot L_{mask}+b\cdot L_{score}+c\cdot L_{box},$$

The same experiment strategy presented in [39] is adopted to define the samples, and the parameters are set as b=c=1 and a=32. Moreover, in this paper, the RoW is considered positive (i.e., $y_{n}=1$) when its anchor box has an IOU with the ground-truth box of at least 0.6 and negative (i.e., $y_{n}=-1$) otherwise.

#### 3.2.4. Box Generation

Different from in the laboratory test, the cable features change drastically in the field test (as shown in Figure 4), which is mainly caused by the complicated structure of the catenary system. In this paper, a simple method is applied to enhance the identification effect of the moving cable, which is called the minimum bounding rectangle. The strategy is used to generate the bounding box from the binary mask, which can be rotated automatically to fit the contact wire shape.

#### 3.2.5. Training and Dataset

The experimental environment is as follows: Ubuntu 14.04 64-bit operating system, 12.0-GB RAM, Intel^®^ Xeon^®^ CPU E3-1230 V2 clocked at 3.30 GHz and GTX 1060 GPU with 8-GB memory. The exemplar and the search images patched in this paper are set as 127×127 and 255×255, respectively. The dataset used consists of contact wire images at different motion states, which are captured from a series of catenary free vibration tests.

### 3.3. The Framework of the Contact Wire Vibration Measurement

In this paper, the proposed devices and method are developed for research on pantograph-catenary dynamics. Given the difficulty in conducting validation on an operating railway line, the field measurement was performed on a catenary testing line, which was built according to the China railway standard TB 10621—2014 and equipped with a T-type pantograph acting as the moving excitation.

**Figure 10:**
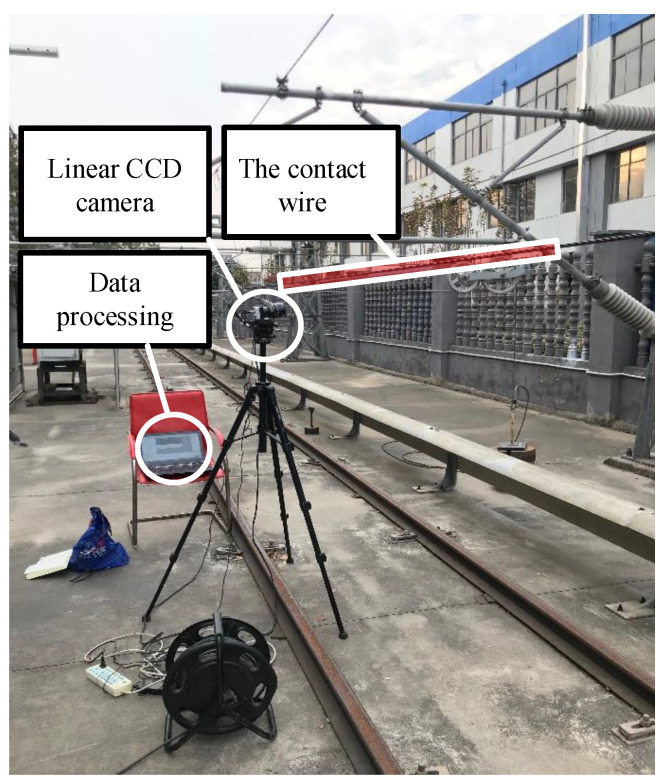
Setup for the field experiment of catenary vibration measurement.

**Figure 11:**
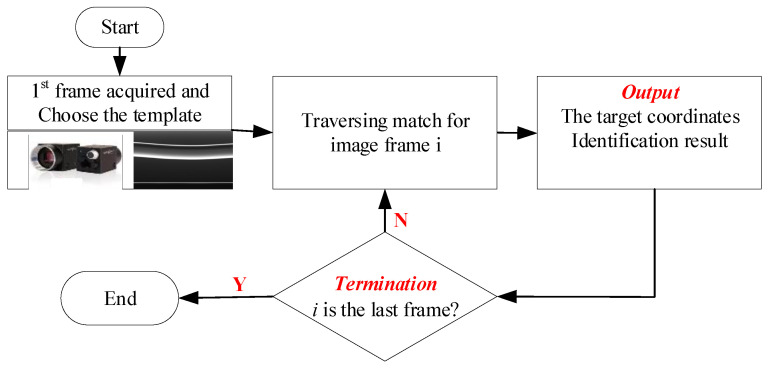
The framework of the proposed method for contact wire vibration measurement under different test conditions.

The verification setup of the proposed method is shown in Figure 10. The devices in the experiment were installed as illustrated in Figure 4. The observing position was placed in the middle between dropper 6 and dropper 7. The image sampling rate was set as 50 frames per second, which was determined considering the field illumination condition, and camera calibration was conducted based on [25]. In this paper, three kinds of vibration cases are taken into consideration: A laboratory test with an illumination supplement, a field test under a weak lighting condition, and a field test under a strong lighting condition. The framework of the measurement with the proposed method is shown in Figure 11, where the coordinates of the contact wire and the image identification results are taken as the final outputs for further analysis.

## 4. Validation through a Field Test

### 4.1. Identification Results Analysis

**Figure 12:**
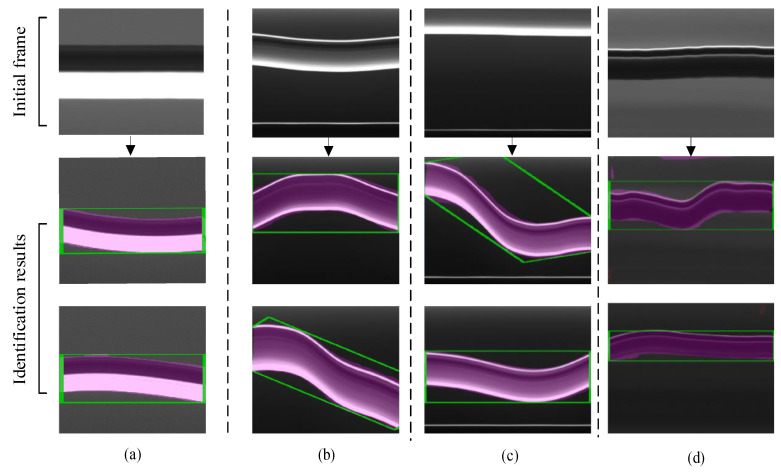
Contact wire vibration images obtained under different conditions. (**a**) Contact wire images obtained from the laboratory test. (**b**,**c**) Contact wire images from the field test under strong lighting conditions and different kinds of impact. (**d**) Contact wire images obtained from the field test under the weak lighting condition.

The catenary vibration identification results obtained via the proposed method are shown in Figure 12. In order to verify the performance of the proposed method for locating and tracking the contact wire, the cable vibration images under different test conditions were taken as the tracking objects. It can be seen that, although the shape and the grayscale value of the vibrating cable rapidly change, the proposed method still displays an acceptable performance in contact wire identification.

Table 2 presents the measurement accuracies of different tracking methods, including the method proposed in this paper, the nonlinear tracking method, and the classical RPN method. The nonlinear tracking method in Table 2 is developed based on the traditional image processing algorithm; specifically, it is a combination tracker consisting of a linear tracker and a nonlinear tracker. The data in Table 1 show that the proposed method performs better than the nonlinear tracking method and the classical RPN method, both in the laboratory test and the field test. Notably, for the laboratory test, the identification accuracies of the three measurement methods do not differ significantly. Nevertheless, the proposed method demonstrates significant advantages in comparison to the other two in catenary vibration measurement in the field test, especially for the measurement under a strong lighting condition.

### 4.2. Comparison with the Simulation Results

To further validate the proposed method, the vibration detection results were compared with the simulation results. The simulation model of the catenary system is established based on the modal analysis, which has been widely used [12,17]. The equation of motions of the catenary system can be expressed as follows:

(5) $$M\overset{\cdot \cdot }{Y}+C\overset{\cdot }{Y}+KY=F,$$

where M,C,K, and F are the mass matrix, damping matrix, stiffness matrix, and excitation vector of the catenary system, respectively. $\overset{\cdot \cdot }{Y}$, $\overset{\cdot }{Y}$, and Y are the acceleration, velocity, and displacement vector of the catenary, respectively. An impulse force is applied at the beginning of the simulation, which also works as the excitation in the field experiment.

The structural parameters of the catenary testing line are shown in Table 3, and were obtained from the China railway standard TB 10621—2014 and field measurement results. The simulation results of the catenary in the time domain and frequency domain can be obtained by adopting the parameters in the table.

#### 4.2.1. Comparison of the Frequency Component

**Figure 13:**
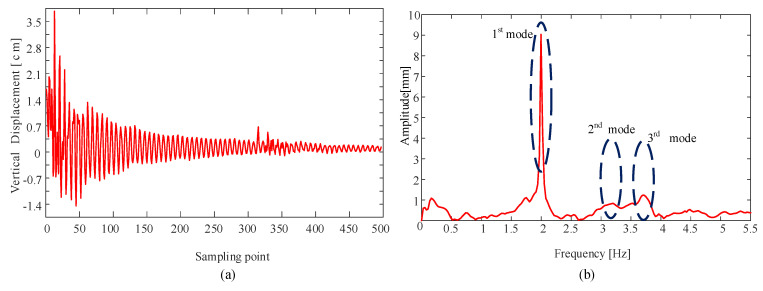
The vibration curve of the contact wire obtained from detection results. (**a**) Displacement of the cable in the time domain. (**b**) Corresponding frequency distribution.

The vertical displacement curve of the contact wire under a strong lighting condition is shown in Figure 13. For the catenary free vibration, the higher energy loss at high frequencies usually leaves the fundamental frequency as the remaining one, as shown in Figure 13, and the first five natural frequencies of the test catenary are calculated using the parameters in Table 3. A comparison of the test catenary frequencies for the calculation and the measurement is shown in Table 4. It can be seen that the three identifiable peak frequencies in Figure 13 are consistent with the first, second, and third natural frequency of the test catenary, respectively.

#### 4.2.2. Comparison in the Time Domain

**Figure 14:**
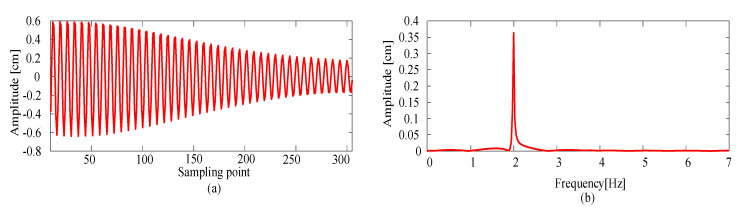
Processed result of the measurement data. (**a**) The vibration curve of the contact wire after removing clutter. (**b**) The corresponding frequency distribution of the processed curve.

Before comparing the data with the simulation results, the measurement data needed to be processed to remove the clutter. Figure 14 depicts the processed result of the measurement data shown in Figure 13, in which only the first-order frequency component remained.

**Figure 15:**
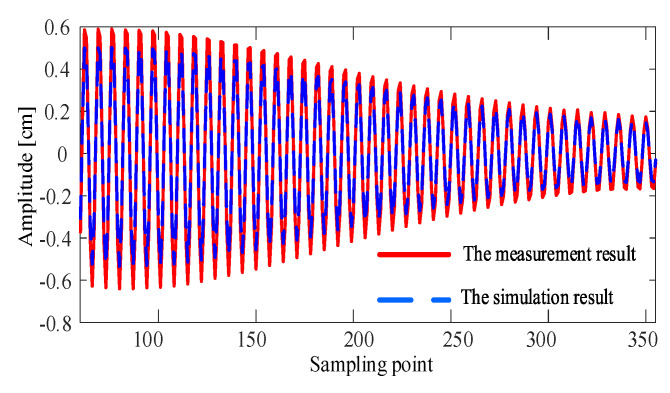
Comparison of the measurement data and simulation data.

Then, the simulation model based on the analytical method was used to obtain the free vibration result of the catenary in the field test. The comparison of the measurement result and the simulation result is shown in Figure 15. The measurement result of the cable free vibration in the field test is consistent with the simulation result. The accuracy of the proposed measurement method is high enough to support the simulation analysis of catenary vibration.

#### 4.2.3. Investigation on the Damping Ratio

It is known that resonance will occur when the excitation frequency is equal to a particular inherent frequency [40]. For a catenary structure, the damping ratio of the system has the effect of reducing and restricting its sustained oscillation. This is why the observed wave gradually disappears as time goes on. The structure damping ratio is a complicated parameter related to material property, vibration frequency, and other factors, and is difficult to identify directly. In this work, the amplitude decay was used to estimate the damping ratio of the catenary testing line. Denoting the damping ratio as ζ and the amplitude of wave peak as φ, ζ can be expressed as follows:

(6) $$\zeta =\frac{1}{2\pi j}\ln\left(\frac{\varphi_{1}}{\varphi_{2}}\cdot \frac{\varphi_{2}}{\varphi_{3}}\cdot \cdot \cdot \frac{\varphi_{j}}{\varphi_{j+1}}\right)=\frac{1}{2\pi j}\ln\frac{\varphi_{1}}{\varphi_{1+j}},$$

**Figure 16:**
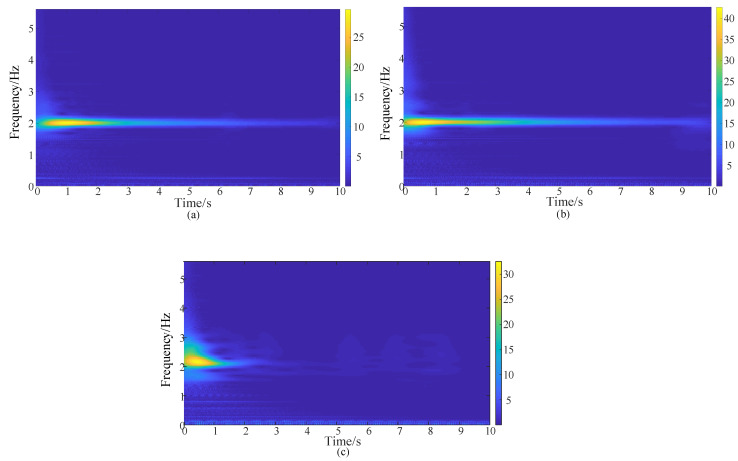
Time-frequency analysis of the extracted signal. (**a**) Excitation at dropper 10, with an uplifted height of 15 cm. (**b**) Excitation at dropper 8, with an uplifted height of 15 cm. (**c**) Excitation at dropper 5, with an uplifted height of 5 cm.

Table 5 gives the damping ratios calculated based on the detection results. Note that all of the damping ratios only correspond to the first frequency component in the signals, as shown in Figure 16. The damping ratios of catenary sections exhibited larger variation with the geometric structure, and also exhibited obvious differences with different measurement distances [22]. From Table 5, it is evident that larger damping ratios occur for larger distances, and the damping ratios change nonlinearly with the change of distances to the excitation position. This discrepancy is due to the fact that the energy of the travelling wave on the catenary will decay with the propagation distance, as well as the dropper number that the wave passed through. To obtain a unique damping ratio related to the first-order frequency, the equivalent value is calculated by considering the weights of observation-excitation distances. The damping ratio of 0.0095 is related to the frequency of 1.997 Hz.

## 5. Conclusions

This paper has proposed a non-contact measurement method using an object tracking strategy for catenary vibration detection. A set of photogrammetric devices were applied in a catenary vibration test in a field environment. The Siamese neural network was used to obtain the contact wire position from field measurement images. The results show that the proposed object tracking method developed in this paper performs well in contact wire vibration identification.

The vibration data obtained from the image identification results are consistent with the simulation results in both the time and frequency domains. The frequency analysis shows that the measurement data have a dominant frequency at around 1.997 Hz, which is the first natural frequency of the test line. In the time domain, the measurement signal is also consistent with the simulation result. In addition, the wave analysis results show that the unique damping ratio of the catenary testing line is determined to be 0.95% related to the frequency 1.997 Hz, which can be used to modify the numerical model and conduct further studies.

Furthermore, in view of the fact that the performance of the proposed method has been tested and validated in this paper, a newly designed field test is currently being conducted on a railway line in operation, in order to obtain the dynamic response of the catenary structure with trains passing.

## Figures and Tables

**Table 1:** Backbone for Resnet_50.

Layer	Kernel	Layer	Kernel
conv 1	7×7, 64, stride 2	conv 4_x	$\left[\begin{array}{l} 1\times 1,\;256\\ 3\times 3,\;256\\ 1\times 1,\;1024 \end{array}\right]\times 6$
conv 2_x	$\begin{array}{c} 3\times 3\;max\;pool,\;stride\;2\\ \left[\begin{array}{l} 1\times 1,\;64\\ 3\times 3,\;64\\ 1\times 1,\;256 \end{array}\right]\times 3 \end{array}$	adjust	1×1, 256
conv 3_x	$\left[\begin{array}{l} 1\times 1,\;128\\ 3\times 3,\;128\\ 1\times 1,\;512 \end{array}\right]\times 4$	xcorr	depth-wise

**Table 2:** Performance of different methods of contact wire vibration measurement under different test conditions.

Detection Method	Working Conditions	Accuracy of IDENTIFICATION (%)
The proposed method	Laboratory test	99.17
	Field test under weak lighting condition	97.42
	Field test under strong lighting condition	96.80
The nonlinear dynamic tracking	Laboratory test	95.83
	Filed test under weak lighting condition	94.32
	Field test under strong lighting condition	88.00
The classical RPN [32]	Laboratory test	94.17
	Field test under weak lighting condition	93.02
	Field test strong lighting condition	75.50

**Table 3:** The structural parameters of the catenary test line.

Index	Value	Index	Value
Length of catenary	69 m	Span number	5
Tension of messenger wire	15 kN	Tension of contact wire	12.75 kN
Type of messenger wire	BZII120	Type of contact wire	CuMg 0.5AC 120

**Table 4:** The natural frequencies (Hz) of the test catenary system.

Order	1st	2nd	3rd
Simulation result	1.998	3.397	3.587
Detection result	1.997	3.235	3.642
Error	0.05%	4.77%	1.53%

**Table 5:** Damping ratio obtained from the identification results.

Observation Position	The Middle between Dropper 6 and Dropper 7		
Excitation position	Dropper 10	Dropper 8	Dropper 5
Uplifted height	15 cm	15 cm	5 cm
Distance to excitation	18.6 m	9.5 m	7.5 m
Damping ratio	0.0125	0.0088	0.0073
Equivalent damping ratio	0.0095		
